# Development and Evaluation of BenchBalance: A System for Benchmarking Balance Capabilities of Wearable Robots and Their Users

**DOI:** 10.3390/s22010119

**Published:** 2021-12-24

**Authors:** Cristina Bayón, Gabriel Delgado-Oleas, Leticia Avellar, Francesca Bentivoglio, Francesco Di Tommaso, Nevio L. Tagliamonte, Eduardo Rocon, Edwin H. F. van Asseldonk

**Affiliations:** 1 Department of Biomechanical Engineering, University of Twente, 7522 NB Enschede, The Netherlands; e.h.f.vanasseldonk@utwente.nl; 2 Centro de Automática y Robótica, Universidad Politécnica de Madrid, 28500 Madrid, Spain; gabriel.delgado@csic.es (G.D.-O.); leticiamunhozavellar.lm@gmail.com (L.A.); e.rocon@csic.es (E.R.); 3 Università Campus Bio-Medico di Roma, 00128 Rome, Italy; francesca.bentivoglio1@gmail.com (F.B.); ditomfrancesco@gmail.com (F.D.T.); n.tagliamonte@hsantalucia.it (N.L.T.); 4 Fondazione Santa Lucia, 00179 Rome, Italy

**Keywords:** balance, assessment, exoskeletons, benchmarking

## Abstract

Recent advances in the control of overground exoskeletons are being centered on improving balance support and decreasing the reliance on crutches. However, appropriate methods to quantify the stability of these exoskeletons (and their users) are still under development. A reliable and reproducible balance assessment is critical to enrich exoskeletons’ performance and their interaction with humans. In this work, we present the BenchBalance system, which is a benchmarking solution to conduct reproducible balance assessments of exoskeletons and their users. Integrating two key elements, i.e., a hand-held perturbator and a smart garment, BenchBalance is a portable and low-cost system that provides a quantitative assessment related to the reaction and capacity of wearable exoskeletons and their users to respond to controlled external perturbations. A software interface is used to guide the experimenter throughout a predefined protocol of measurable perturbations, taking into account antero-posterior and mediolateral responses. In total, the protocol is composed of sixteen perturbation conditions, which vary in magnitude and location while still controlling their orientation. The data acquired by the interface are classified and saved for a subsequent analysis based on synthetic metrics. In this paper, we present a proof of principle of the BenchBalance system with a healthy user in two scenarios: subject not wearing and subject wearing the H2 lower-limb exoskeleton. After a brief training period, the experimenter was able to provide the manual perturbations of the protocol in a consistent and reproducible way. The balance metrics defined within the BenchBalance framework were able to detect differences in performance depending on the perturbation magnitude, location, and the presence or not of the exoskeleton. The BenchBalance system will be integrated at EUROBENCH facilities to benchmark the balance capabilities of wearable exoskeletons and their users.

## 1. Introduction

Wearable robotic devices such as lower-limb exoskeletons have attracted extensive interest in the last decades, demonstrating the ability to support people with motor impairments in standing and walking. Recent research on these devices is moving toward the development of controllers to assist balance during standing and/or walking. These controllers may involve different degrees of freedom (DOFs) to improve exoskeleton-user stability and may vary in complexity, using (1) control strategies derived from humanoids applications [1,2,3,4], (2) bio-inspired approaches [5,6,7], or (3) simple heuristic methods [8,9,10]. However, appropriate procedures and metrics to properly quantify, compare, and assess the stability of different exoskeletons, controllers, and their users, as well as the overall combination of them, are still scarcely defined.

Current approaches to measure the balance performance of a single wearable robot are mainly based on changes in the human’s motor function [11]. From there, there is still a big step to achieve the final goal of new standards for benchmarking on a vast scale [11,12]. A possible starting point toward developing objective standards of wearable robot balance skills is to first consider the different developments in balance assessment of humans (without exoskeletons). Later, these assessment tests can be extended to wearable devices and their users. Balance assessments in humans may be classified into three principal approaches [13]: (1) functional assessments; (2) systems and physiological assessments; and (3) quantitative assessments. Regarding the latter, Shirota et al. [14] presented an overview of assessments using robotic devices and technologies. This review included not only the use of conventional quantitative methods such as posturography [15] or unobtrusive sensors during clinical procedures [16] but also other novel technologies suitable for balance assessment.

One class of these devices is extensively used to test the ability of balance control by applying a controlled external disturbance such as a push against the body [17,18,19]. However, these robotic devices are generally expensive, complex, and present several limitations when being used with human wearing wearable devices: for instance, problems with the fixation of the testing device to the human body when a wearable exoskeleton is being worn. Moreover, to the best of our knowledge, all the existing solutions are restricted to even-ground conditions and present a limited versatility to be used in relevant environments outside the laboratory. Summarizing, the possibility of applying perturbations with this type of device in challenging environments or at different locations of the human body is limited, especially if the user wears an exoskeleton.

To address the challenges of performing balance tests with people wearing an exoskeleton, simpler and movable devices based on strain gauges were developed [20]. This type of push stick can measure the magnitude of perturbations manually applied by the experimenter to the human body. However, it still has some limitations such as bulkiness, restriction to wired connections, and limited structural robustness. Furthermore, the location and the orientation of the force applied to the human body cannot be measured with these push sticks, being difficult to provide an objective feedback to the experimenter and thus limiting the ability to control whether the perturbations are being provided in a consistent way.

**Figure 1:**
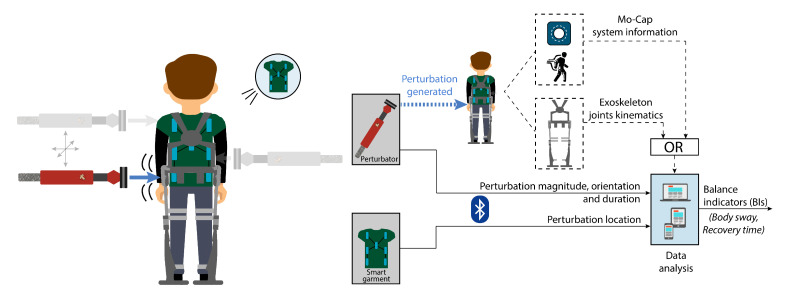
Overview of the BenchBalance testbed. The acquired signals are transmitted via Bluetooth to the processing unit for its analysis.

New methods are needed to compare users’ and wearable robots’ performances using quantitative and reliable metrics. The main goal of this work is to improve the current methods to assess balance capabilities of wearable exoskeletons and their users by proposing a novel benchmarking solution that is able to conduct reliable assessments under reproducible perturbed conditions in both stationary and challenging environments. Our system, BenchBalance (Figure 1), is a portable solution capable of measuring well-defined external perturbations manually provided by an experimenter, in terms of magnitude, orientation, and location, to subjects wearing (or not) an exoskeleton. The system does not require a fixation to the human body, so the provided perturbations can be generated in any DOF of the three planes of motion, and they can be applied in a wide range of situations and environments. In this contribution, we describe the definition and design of the complete BenchBalance system, its mechanical and control architecture, and the interface and algorithms developed for data processing and derivation of metrics. Finally, as a proof of concept, we test the system with a subject following a protocol of sixteen perturbations (varying in magnitude and location) while wearing and not wearing a lower-limb exoskeleton. The information included in this paper allows a reproduction of the BenchBalance system to other research groups interested in benchmarking perturbed balance.

## 2. Materials and Methods

### 2.1. BenchBalance System

For an accurate balance assessment and to be able to derive quantitative metrics of the balance capabilities of a human wearing (or not) an exoskeleton, we focused on the following requirements:

- Quantify the disturbance applied in terms of force magnitude and orientation;
- Quantify where a perturbation is applied on the human body, since balance recovery strategies might differ depending on the location of such perturbation;
- Ensure an appropriate synchronization of the perturbation with the user’s response;
- Provide real-time feedback to the experimenter to augment the ability of providing perturbations in a consistent way;
- Calculate outcome indicators to quantify the balance response by using kinematic data collected either with any motion capture system (Mocap) or with the on-board exoskeleton sensors.

Based on these requirements, the BenchBalance testbed (Figure 1) is composed of the following: (1) a portable hand-held perturbator equipped with force and orientation sensors, which is used to provide and quantify well-defined pushes to the human upper body during both standing and walking conditions; (2) a position system detector (smart garment), which is used to determine the location of the generated perturbation in relation to the human wearing the exoskeleton; and (3) an analysis unit that combines the information of the provided force (magnitude, direction, and location) with joint angle data from the wearable exoskeleton or Mocap system and generates the Balance Indicators (BIs), i.e., metrics to complete the assessment.

#### 2.1.1. Perturbator

**Figure 2:**
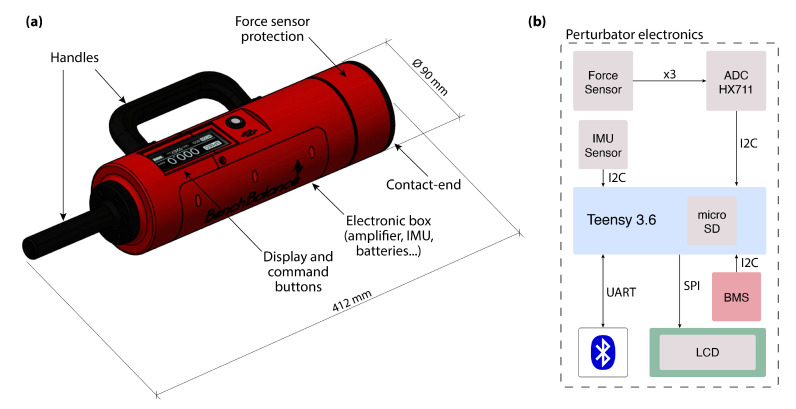
Portable perturbator overview: (**a**) CAD model and elements; (**b**) Electronic connections.

A battery-driven manual perturbator (Figure 2a) was designed to provide and quantify the external disturbances to the user. The frame of the perturbator was made using PLA 3D printing material, which was reinforced with metal in the handles to increase its robustness. The maximum length of the perturbator is 412 mm with a diameter at the contact end of 90 mm. With a total weight (including sensors and electronics) of 1.2 kg, the perturbator can be easily handled by the operator, so the versatility of the device is very high in terms of usability and the ability to apply a wide range of perturbations.

The housing includes a command panel with a graphic display that indicates to the experimenter the most relevant real-time information on the perturbations, an USB connector to charge the battery, and a push button for on/off switching.

##### Electronics and Signal Processing

The perturbator is equipped with two main sensors used to measure the magnitude and the orientation of the perturbation:

- A three-axial force sensor, K3D60a (ME Systeme GmbH, Hennigsdorf, Germany). The nominal force of the sensor is 500 N with an accuracy of 0.2% in all directions, which was a requirement to properly quantify the magnitude of the perturbation applied to the user.
- An inertial measurement unit (IMU) MTi-3 AHRS (Xsens, Enschede, The Netherlands). Together with another inertial sensor integrated in the smart garment subsystem, it is used to estimate the relative orientation of the perturbation with respect to the human.

In addition to the previous main sensors, the electronics of the perturbator (Figure 2b) also include the following:

- Amplifier with ADC 24 bits (HX711), used to measure the force detected by the force sensor.
- LCD display, New Haven, NHD-2.8-25664UMY3, included in the command panel to show relevant real-time information to the experimenter, i.e., perturbation amplitude, impulse, and orientation.
- Micro SD card, used to pre-store the data of the experiment that will be later transferred to the host computer (sampling frequency of data collected: 30 Hz).
- Battery Management System (BMS), to control a one-cell lithium polymer (LiPo) battery (3.7 V, 2000 mAh) used to supply the microprocessor board and the sensors.

A microprocessor board (Teensy 3.6, ARM Cortex-M4 at 180 MHz, PJRC, Sherwood, United States) was used for the communication among all the components of the perturbator (Figure 2b). One thread of the processor was dedicated for Bluetooth transmission, and the second thread was used for the algorithms of the rest of the electronics. The firmware running in the microprocessor is written in C language. The internal communication between the different electronic components was performed by using both serial peripheral interface—SPI (for the LCD display), and inter-integrated circuit interface—I2C (for the force and IMU sensors and the BMS). Both protocols are synchronous, so the outputs of bits were synchronized to the sampling of bits by a clock signal shared between the master (Teensy 3.6) and the slaves.

To calibrate the 3D force sensor, an algorithm was developed, which is being executed every time that a protocol with BenchBalance is performed. During the calibration, 2000 data samples of the three axes are used to calculate the corresponding mean values per axis. The calibration of the IMU is performed similarly, deriving as output signals the quaternions and the Euler angles to indicate the orientation to the experimenter.

To write the raw data in the microSD, we use the SD protocol (SDIO). A Real-Time Clock (RTC) was used for inter-tasks communication, which is automatically calibrated when being connected to the host computer. Bluetooth 3.0 was selected to transfer the collected data in real time to the host computer (30 Hz). The decision was made based on internal requirements to ensure sufficient bandwidth and synchronization.

#### 2.1.2. Smart Garment

**Figure 3:**
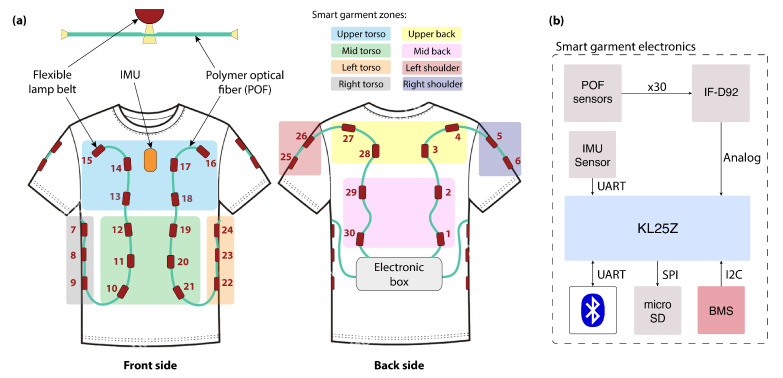
Smart garment overview: (**a**) Components and zones differentiation; (**b**) Electronic connections.

A textile vest instrumented with polymeric optical fibers (POFs) (Figure 3) was designed to detect the area of application of the perturbation in the user wearing the exoskeleton. It offers a robust, minimally invasive, and multiplexed solution to monitor the pressure of the perturbation alongside the human upper body. The smart garment was manufactured in three different sizes, ensuring a broad application and accommodating the size difference between users.

The textile vest is equipped with two embedded POFs made of polymethyl-methacrylate (PMMA-HFBR-EUS100Z, Broadcom, San Jose, United States). Each POF has a circular cross-section with three layers, being from inside to outside: the core, the cladding, and the buffer coating. The total diameter for our POF, including its polyethylene coating, is 2.2 mm, being the diameter of the core equal to 980 µm, and the thickness of the fluorinated polymer cladding equal to 20 µm.

Thirty sensors are distributed along the POFs, covering the front and back area of the trunk, the area below the arms, and the shoulders (Figure 3a). Each sensor has an independent source light (i.e., a light-emitting diode, LED), and each end of the POF has a photodetector (four in total) located in the electronic box placed on the back part of the garment. To increase the sensitivity of the sensors and to enable the side coupling of the light source, we followed the guidelines presented in [21].

The measuring principle for these sensors is based on light intensity variation, which is done by converting the light intensity provided by the LED into voltage. The multiplexing technique proposed by Leal-Junior et al. [22] was used to decouple the sensor’s individual response and thereby enable knowing in which area of the smart garment the perturbation was being provided. This technique is based on the sequential activation of each LED with a predefined frequency and activation sequence.

##### Electronics and Signal Processing

The main sensors that compose the smart garment are the above-mentioned thirty POF sensors, together with an IMU sensor (Xsens—MTi-3 AHRS), which is integrated in the textile and has the same characteristics than the one of the portable perturbator. This IMU is used to measure the orientation of the human body. Thereby, by using both the IMU of the perturbator and the one of the smart garment, we can derive the relative orientation of the provided perturbation with respect to the human.

For the communication of the electronic components of the smart garment, we used a microprocessor board (NXP FRDM-KL25Z, Figure 3b). Similarly to what was done for the perturbator, the first thread of the microprocessor was dedicated to Bluetooth transmission and the second thread dedicated to the communication of the rest of electronics, including the following:

- Four high-sensitive phototransistor detectors (Industrial Fiberoptics, IF-D92) to transform the light intensities into voltages.
- Micro SD card module to pre-store the data collected during the experiment that later will be transferred to the host computer (sampling frequency of data collected: 30 Hz).
- BMS (SparkFun Battery Babysitter) to monitor a LiPo battery (3.7 V, 2000 mAh), which is used to supply the microprocessor board and the sensors.

As done for the perturbator, the communication between the different electronic components of the smart garment was performed via SPI and I2C interfaces. Concretely, the Micro SD card was connected in this case via the SPI bus and the BMS via I2C bus. The IMU sensor was connected to the KL25Z via UART. The phototransistor detectors (IF-D92) were coupled to the ADC (16 bits) ports of the microprocessor.

To calibrate the thirty POF sensors, 200 data samples are taken from each of them and used to set threshold values to indicate the zero activation. A sensor is being recognized as active when its threshold value is being overpassed by pressing the sensor. During the use of BenchBalance, the four most active sensors within each data sample are being sent in a pack of data to the host computer.

For the IMU of the smart garment, we follow the same calibration procedure than with the IMU of the perturbator, calculating as well the quaternions and Euler angles. The global coordinate frames for both IMUs are also obtained during the calibration of the whole BenchBalance system.

The raw data of the smart garment are transferred in real time using Bluetooth 3.0 to the host computer and synchronized with the raw data of the perturbator by timestamps.

### 2.2. Data Recording and Metrics Derivation

#### 2.2.1. User Control Interface

**Figure 4:**
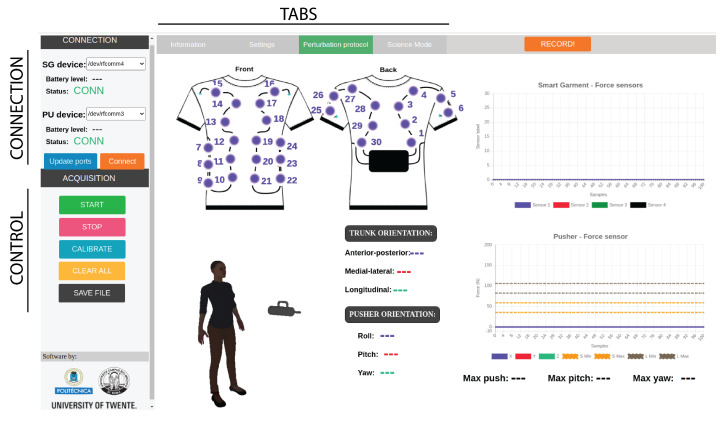
User interface to control balance assessment with BenchBalance device.

To facilitate the use of the BenchBalance system, we developed an acquisition software interface (Figure 4), which acquires the data of the perturbator and the smart garment and guides the experimenter throughout the required steps to deliver reproducible perturbations to the human body. This interface is programmed in JavaScript–HTML, and it includes all the requirements to follow a pre-defined protocol of perturbations to assess the user’s balance in both antero-posterior (AP) and mediolateral (ML) directions.

Before starting the measurements, it is required to calibrate the system to remove offset values and define the coordinate frames. This is done through the interface, which indicates to the experimenter how to put the perturbator and smart garment in the calibration position and for how long until the offset values have been removed. After calibrating the system, the experimenter should insert the anthropometric properties (height, total mass) of the test subject and indicate the presence or not of the exoskeleton, including its mass. These properties are used by the interface to calculate the tolerances of the perturbation magnitudes to be applied in the pre-defined protocol. The data recorded during the protocol are classified and saved at a host computer. It allows the posterior comparison between different recordings performances in a standardized way.

#### 2.2.2. Controlled Variables and Protocol

**Figure 5:**
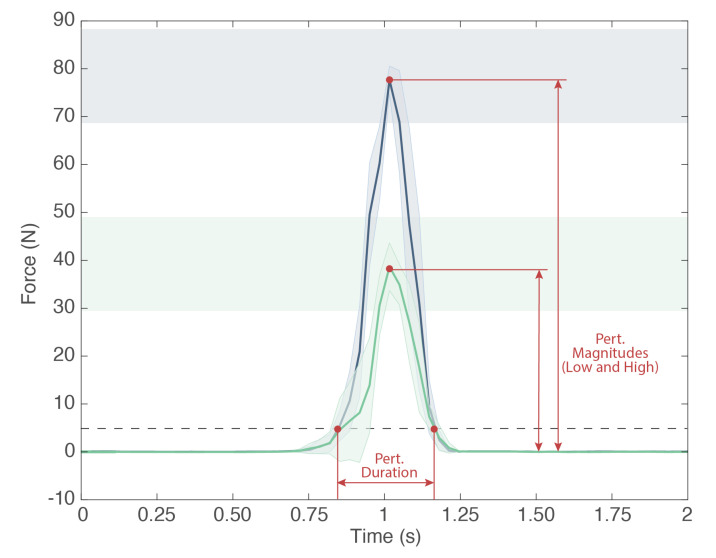
Example of mean and SD of five suitable perturbations provided for Low (green) and High (blue) magnitudes. The perturbation duration is defined as the interval in which the force is exceeding the detection threshold, which is set to 5 N (dashed line).

**Figure 6:**
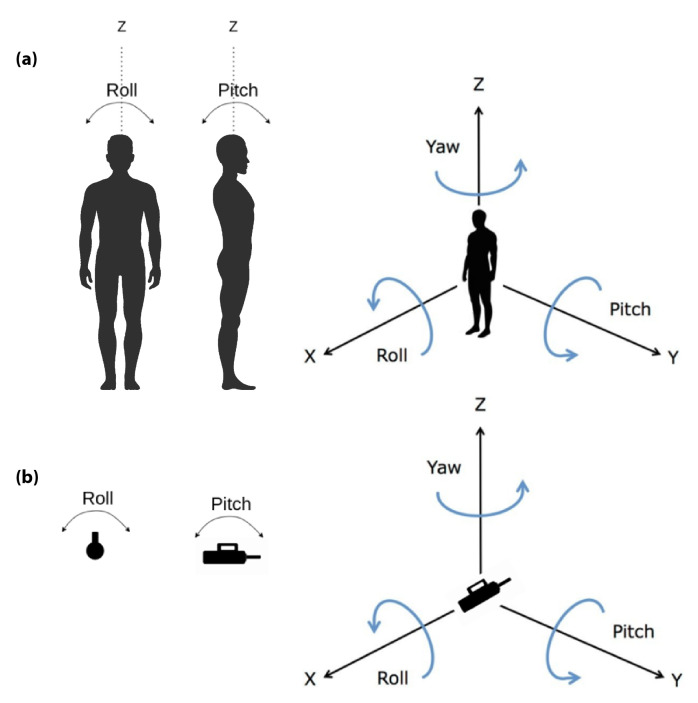
Reference frames for (**a**) the smart garment on the human and (**b**) the perturbator.

Controlled variables (CVs) refer to parameters and features that are monitored by the acquisition interface to guarantee homogeneous assessment conditions. To account for different kinds of perturbations (magnitude, orientation, duration, and location) in both AP and ML directions, we defined a protocol with sixteen conditions for the standing balance (see Table 1). The CVs evaluated within this protocol include the following:

- Perturbation magnitude: It is the maximum amplitude of the force (N) applied by the experimenter to the subject by means of the perturbator. In particular, the perturbator monitors the forces along all three axes, and we calculate the magnitude of the resultant vector. Within the BenchBalance protocol (Table 1), we consider two levels of perturbation magnitude: “Low”, computed as 8 ± 2% of the total mass (body mass plus mass of the exoskeleton, if present); and “High”, computed as 16 ± 2% of the total mass (Figure 5).
- Perturbation duration: It is the time interval along which the force is applied by the experimenter by means of the perturbator. It is calculated as the elapsed time of the force exceeding a (“no force”) threshold of 5 N, and it is expressed in seconds (Figure 5). For the BenchBalance protocol, an acceptable value of the perturbation duration is 0.35 ± 0.15 s for all conditions.
- Perturbation orientation: It is the relative orientation between the human upper body and the direction of the force vector applied to the subject. It is measured by means of the IMUs of the smart garment and the perturbator, and it is expressed as pitch, yaw, and roll components (see reference frames in Figure 6). Within the BenchBalance protocol, we consider acceptable perturbations applied perpendicular to the human body with a tolerance of ±30 degrees in pitch and yaw angles for all conditions.
- Perturbation location: It is the area of the subject’s upper body where the force is applied. This location is extracted by checking the number of the four most active sensors in the smart garment, identifying them based on the zones reported in Figure 3.
- Exoskeleton: It is a Boolean value indicating the presence or not of the exoskeleton in the experiment (0: user without exoskeleton; 1: user with exoskeleton). If an exoskeleton is present, its characteristics (dimensions and mass distribution) are considered for BIs calculation, as described in the following section.

**Table 1:** Location and type of the perturbations within the pre-defined protocol. The selection of sensors is based on the zones defined in Figure 3. M stands for the total mass of the participant (+ exoskeleton if present). Five good repetitions of the same condition are required to be recorded by the user interface.

Condition Order	Perturbation Location	Sensors	Perturbation Magnitude	Perturbation Type
1	Mid back	1–2–29–30	8 ± 2% M	Low
2	Upper back	3–4–27–28	8 ± 2% M	Low
3	Mid back	1–2–29–30	16 ± 2% M	High
4	Upper back	3–4–27–28	16 ± 2% M	High
5	Mid torso	10–11–12–19–20–21	8 ± 2% M	Low
6	Upper torso	13–14–15–16–17–18	8 ± 2% M	Low
7	Mid torso	10–11–12–19–20–21	16 ± 2% M	High
8	Upper torso	13–14–15–16–17–18	16 ± 2% M	High
9	Right side torso	7–8–9	8 ± 2% M	Low
10	Right side shoulder	5–6	8 ± 2% M	Low
11	Right side torso	7–8–9	16 ± 2% M	High
12	Right side shoulder	5–6	16 ± 2% M	High
13	Left side torso	22–23–24	8 ± 2% M	Low
14	Left side shoulder	25–26	8 ± 2% M	Low
15	Left side torso	22–23–24	16 ± 2% M	High
16	Left side shoulder	25–26	16 ± 2% M	High

If during the execution of the protocol, any perturbation occurs that does not comply with the previous CVs, the acquisition interface generates a flag indicating in real time to the experimenter that the provided perturbation was not correct, and thereby, it needs to be repeated. This flag is also stored in the saved data for the post-processing.

#### 2.2.3. Balance Indicators (BIs)

The BenchBalance algorithms calculate two BIs: (1) body sway and (2) recovery time, which are commonly used indicators to assess perturbed balance during stance [20,23].

**Figure 7:**
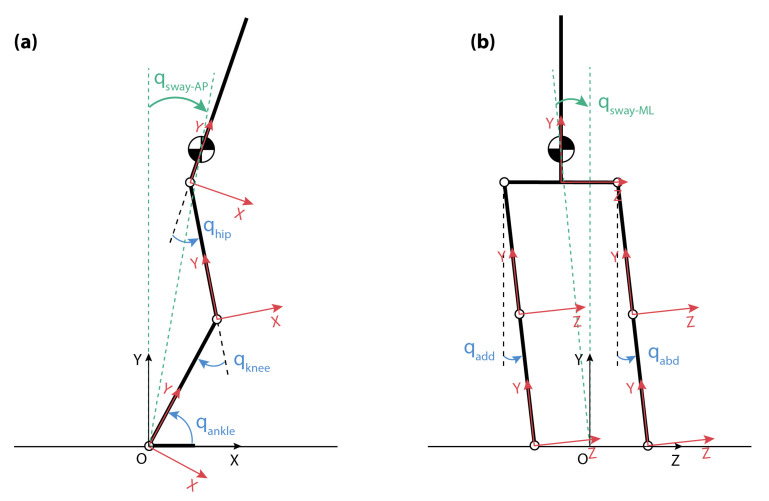
Simplified (**a**) sagittal and (**b**) frontal model used in the calculation of the BenchBalance performance indicators. The convention for joint angles ($q_{hip}$, $q_{knee}$, $q_{ankle}$, $q_{add/abd}$, in blue) and sway angle $q_{sway}$ (green) are indicated for both planes.

These BIs are derived from the kinematic data of the combined (human + exoskeleton) Center of Mass (CoM), which was calculated based on a simplified 2D model defined for both sagittal and frontal planes (Figure 7). This type of model is a well-known approach widely used in the literature for the analysis of postural reactions during stance [4,7,20,23,24,25]. In our case, it is composed of a 5-segment rigid body system: bilateral shank segments, bilateral thigh segments, and HAT (head, arms, and trunk). The inter-segment connections were considered to be pure rotatory joints. The feet were assumed to be flat on the ground; i.e., the ankle joint was virtually connected directly with the ground.

Two parts of the model were defined for the analysis (Figure 7): (1) a sagittal plane model to account for AP perturbations; and (2) a frontal plane model to account for ML perturbations. Some assumptions were considered:

- Left and right legs are considered identical and symmetrically placed.
- Only ankle, knee, and hip pure flexion/extension angles were considered for the sagittal plane model, and only pure hip add/abduction was used to define the orientation for the frontal plane model. Each joint angle was derived by comparing the relative orientations of the proximal and distal segments around each joint.
- Left/right angles were considered equal (left/right average of collected data).

As input parameters for the BenchBalance model, we required the following: (1) the length of the human segments (thigh, shank, and HAT [26]), which can be directly measured on the subject’s body; (2) the masses and the CoM position of each segment, which are computed from the total subject mass by using approximations from standard anthropometric data [27]; and (3) the mass properties and the CoM positions of the single links of the exoskeleton, if present, to be combined with those of the human.

The CoM of the human ($CoM_{H}$) and the CoM of the exoskeleton ($CoM_{E}$, when the exoskeleton was present) were calculated separately by using the defined model with the assumption that human segments and exoskeletons links had exactly the same length, and hence, human and robotic joints were perfectly aligned. Subsequently, the total (human+exoskeleton) center of mass $CoM_{T}$ was calculated as follows:

(1) $$CoM_{T}=\frac{CoM_{H}M_{H}+CoM_{E}M_{E}}{M_{H}+M_{E}}$$

where $M_{H}$ and $M_{E}$ are the masses of the human and exoskeleton, respectively. Note that if the exoskeleton is not present, $CoM_{T}$ = $CoM_{H}$.

The computation of the CoM motion performed with the BenchBalance sagittal/frontal 2D model was validated by comparing it with a reference estimation computed by means of a commercial IMU-based motion capture system and its biomechanical software (Xsens MVN, Enschede, The Netherlands). This reference measurement system uses a full human tracking composed by 17 IMUs attached to different body segments, which estimate the 3D position and orientation of these segments. The positions and orientations, together with a body mass distribution model, result in the calculation of the total body CoM [28]. Representative data demonstrating the consistency between the BenchBalance model and the calculation from the full-body 3D mocap system model are reported in Appendix B.

For every perturbation that complied with the above-mentioned CVs, we used the $CoM_{T}$ to calculate the corresponding BIs. In the calculation of BIs, the model of BenchBalance only uses joint angle data besides the anthropometric measures of the subject. Hence, even if the model is simplified, it is extremely versatile, since it can be applied to several experimental conditions in which joint angles can be measured in different ways, e.g., by IMU-based sensors, optoelectronic cameras, or angular sensors embedded in exoskeleton joints.

The two BIs of BenchBalanceare are calculated as follows:

- Body sway ($q_{sway}$): It represents the maximum body angle in response to a perturbation. A high value of the $q_{sway}$ indicates less ability of the subject in maintaining balance. This BI was evaluated separately for AP and ML (Figure 7a,b). Considering the Cartesian coordinates of the combined $CoM_{T}$ ($CoM_{T}^{x}$, $CoM_{T}^{y}$, $CoM_{T}^{z}$), $q_{sway}$ was calculated as:
  (2) $$\left\{\begin{array}{c} q_{sway-AP}=arctan\left(\frac{CoM_{T}^{x}}{CoM_{T}^{y}}\right)\\ q_{sway-ML}=arctan\left(\frac{CoM_{T}^{z}}{CoM_{T}^{y}}\right). \end{array}\right.$$
- Recovery time: It stands for the time spent to recover from a perturbation, i.e., the time needed to get the CoM back to the rest position within a certain error tolerance. Here, we consider that the position is being recovered if the sway velocity is lower than a threshold of 0.86 deg/s for at least 0.5 s after the perturbation onset.

### 2.3. Proof of Principle

To provide an example of the functional capabilities, data analysis, and derivation of metrics of the BenchBalance system, a proof of principle is reported (see movie on Video S1 and the system setup in Appendix C).

A healthy female participant (age 31 years old, weight 60 kg, height 1.76 m) was selected as the test subject. The BenchBalance system was tested in two scenarios: (1) participant without exoskeleton; and (2) participant wearing the H2 exoskeleton with hip, knee, and ankle actuators in passive mode (i.e., not active) [29]. In both scenarios, the participant wore the smart garment of BenchBalance and a Xsens full body motion capture suit (Xsens, Enschede, The Netherlands). The latter was used to record full body kinematics [28] and provide the joint angles that were later employed to extract the CoM motion, the sway angle ($q_{sway}$), and its velocity by means of the BenchBalance model (Figure 7). The Xsens suit was calibrated separately for each scenario, and the recorded data were synchronized with the BenchBalance data by means of a trigger signal received by both systems.

During the experiment, the participant was asked to stand with feet shoulder width apart and informed that she would receive perturbations with different intensities and locations (following the protocol presented in Table 1). The task of the participant was to counteract these perturbations without taking a step or lifting the heels or the toes.

The perturbations were manually delivered by an experimenter by means of the BenchBalance perturbator. Consecutive perturbations were separated by at least three seconds to guarantee that the participant had recovered the initial standing position between perturbations.

The software acquisition interface of BenchBalance provided visual and audio feedback to inform the experimenter whether a perturbation was acceptable, i.e., within the selected tolerance ranges defined in Section 2.2.2, or needed to be repeated. Five acceptable repetitions of the same desired perturbation were required to be collected.

## 3. Results

### 3.1. Performance of the BenchBalance System

The experimenter was able to familiarize with the BenchBalance system and could provide the perturbations defined in the protocol within 20 min. The system was accurate in assessing the magnitude, duration, orientation, and location of the perturbations and in giving feedback to the experimenter to keep the perturbations within the pre-defined tolerance ranges.

**Figure 8:**
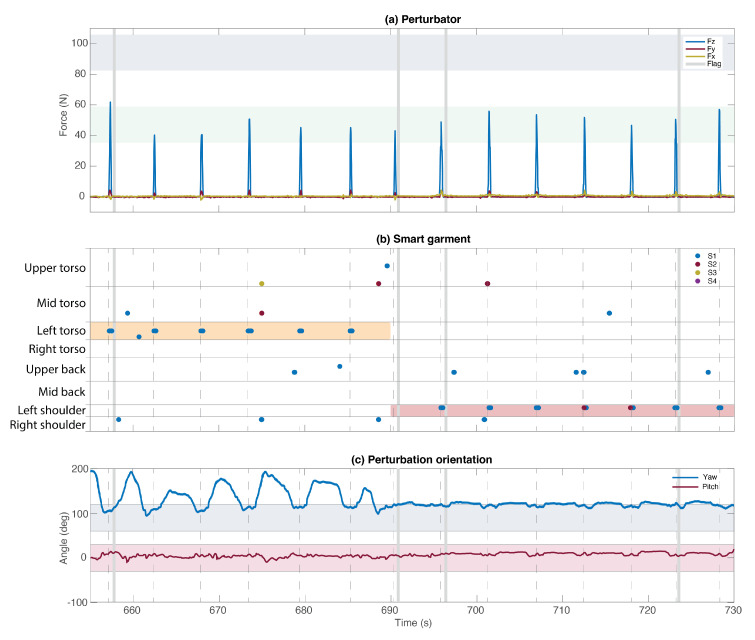
Data extracted from the experiment where the participant was not wearing the exoskeleton. The execution of conditions 13 (left side torso—Low) and 14 (left side shoulder—Low) is represented. The pre-defined tolerance ranges for the magnitude, location, and orientation are represented by the colored areas: (**a**) Perturbator—Low perturbations area in green, and High perturbations area in blue; (**b**) Smart garment—left torso area in orange, and left shoulder area in red; (**c**) Yaw angle area in blue, and pitch angle area in red. On the (**b**) Smart garment subplot, S1 to S4 refer to the four most active sensors registered at a time. At least one active sensor must be within the targeted area of the garment to comply with the location of the perturbation. Vertical gray bars represent the flag provided by the BenchBalance system to identify the discarded perturbations due to non-compliance with any of the tolerance ranges of magnitude, duration, location, or orientation.

Figure 8 demonstrates an example of how the system checked whether the perturbations were applied correctly. If during the execution of a specific condition of Table 1, the provided perturbation did not comply with the pre-defined tolerance ranges of magnitude, duration, location, or orientation, the system detected it (vertical gray bars in Figure 8) and provided the corresponding audio and visual feedback to the experimenter. Once (and only if) five adequate perturbations were provided within the same condition, the acquisition interface of BenchBalance indicated to the experimenter to move to the next condition of the protocol.

### 3.2. Participant and Exoskeleton Performances

The participant’s balance reactions to the provided perturbations were assessed by the BIs of BenchBalance by using the simplified models presented in Figure 7. Repetition averages and standard deviations (SD) of reactions were computed by using the five adequately delivered perturbations for each condition of the protocol.

**Figure 9:**
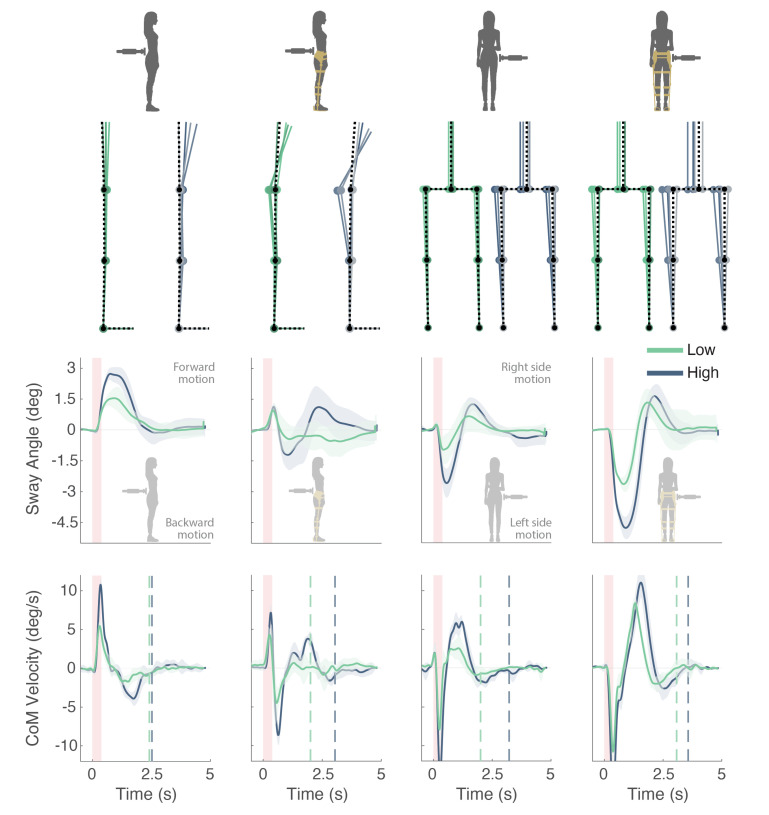
Example of participant’s performance in two scenarios (not wearing and wearing the exoskeleton) and four perturbation conditions (mid back—Low and High, and right side torso—Low and High). In all figures, the mean ± SD across the five adequate perturbations applied for each condition are presented, with responses to Low perturbations in green, and responses to High perturbations in blue. The stick diagrams of the first row simulate the averaged movement performed by the participant and exoskeleton in each condition. The second and third row show the time-series data of body sway angle and CoM velocity respectively, which are used to calculate the BIs. The onset and duration of the provided perturbations are indicated by the red vertical areas, while the vertical dotted lines represent the recovery times for each condition.

**Figure 10:**
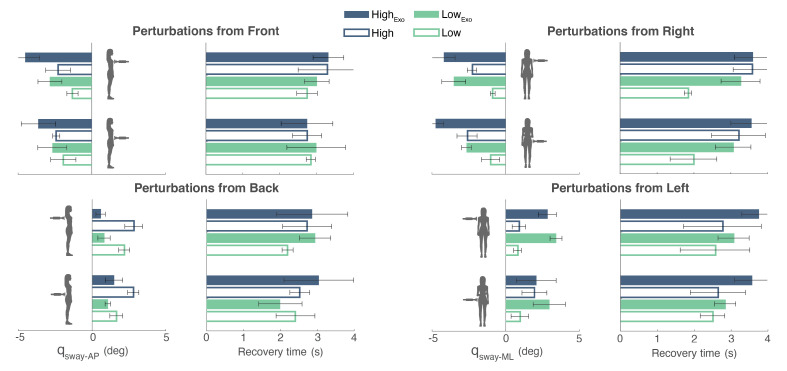
BIs calculation (mean ± SD) for all conditions of the protocol in both scenarios (exoskeleton present—filled bars; and exoskeleton not present—empty bars). Low and High magnitudes of perturbations are presented in green and blue, respectively. Note that for frontal and right-side perturbations, the $q_{sway}$ is negative, as it was established in the sign convention of Figure 7.

The BIs of BenchBalance were able to detect and report differences in balance performance depending on the perturbation magnitude and the presence or not of the exoskeleton (Figure 9). In the majority of the conditions, both the $q_{sway}$ and the recovery time were larger with the High perturbation magnitude than with the Low one (see Figure 10 and Table A1 reported in Appendix A). Additionally, when the participant wore the exoskeleton, balance reactions involved larger joint excursions than when the exoskeleton was not present. In general, the reactions with the exoskeleton included hip and ankle balance strategies (stick diagrams in Figure 9), normally resulting in larger $q_{sway}$ and recovery times.

The response of $q_{sway}$ with and without the exoskeleton had the same pattern but with a different amplitude for all perturbation conditions except for the perturbations from the back (Figure 9 and Figure 10). For the perturbations from the back, the response of the participant was dominated by a different balance strategy when she was wearing or not wearing the exoskeleton. When not wearing the exoskeleton, the total $CoM_{T}=CoM_{H}$ (and thereby the $q_{sway}$) described a forward movement in AP direction due to the flexion of the trunk. However, when the participant wore the the exoskeleton, despite the flexion of the trunk, the combined $CoM_{T}$ did not generate a large positive value in AP, which might be because of two reasons: (1) the $CoM_{E}$ was behind the subject; (2) the participant also bent the knees to counteract the perturbation. This divergence in balance responses with/without the exoskeleton was more evident for High than for Low perturbation magnitude.

## 4. Discussion

An objective assessment of postural and balance control of exoskeletons and their users is indispensable to create a solid and unified benchmarking of wearable robots [11,30]. In the present contribution, we described in detail the hardware and software of the BenchBalance system, which is a novel solution to assess and compare the balance capabilities of users wearing or not an exoskeleton.

To assess and compare these balance capabilities, the definition of performance metrics was required. A straightforward metric might be to account for the ability to tolerate the provided external disturbances, i.e., the maximum perturbation magnitude that the user + exoskeleton are able to successfully stand can be registered as a score. However, it is not safe or recommendable to “push until failure”. Instead, we agreed upon the definition of other performance metrics that provided an interpretation of the balance capability in terms of the reaction to moderate perturbations. In that sense, BenchBalance projects includes two BIs: the evoked body CoM sway and the time needed to recover from the perturbation [20,23].

One of the main advantages of the BenchBalance system is that it is a low-cost and portable solution that gives a high versatility to the experimenter compared to other existing approaches, which consist of robotic devices that are complex and stationary, requiring structured experimental environments [17,18,19]. Its use is not restricted to even-ground laboratory conditions, which opens a wide range of options to assess balance in unstructured and challenging environments. Additionally, the use of BenchBalance does not require a fixation of the device to the human, as previous approaches do. This means that our system provides freedom to apply perturbations at different locations of the human body and in different directions.

Due to the fact that the perturbator of BenchBalance is controlled manually, it might require practice to become proficient in using it in order to keep the controlled variables within the tolerance ranges. To tackle this potential issue, we created a user-friendly acquisition interface, which instructed and gave feedback to the experimenter, making it easier to provide the pre-defined perturbations of the protocol. By using this interface, the experimenter of the test present study could learn to operate the system in less than 10 min, being able to finish the full protocol of perturbations performed in the present study within 20 min.

In the conducted experiment, we focused on a two-dimensional static balance assessment while keeping the feet in place and flat to the ground. This approach has been extensively used in the literature by other groups using similar simplified models of inverted single, double, or triple pendulum [4,7,20,23,24,25]. Future extensions of the model used in BenchBalance will include both the measurement of the foot angles and the consideration of balance control as a three-dimensional problem. These advances, together with the portability of the system, will allow us to extend the calculation of the BIs to broader scenarios as the assessment of balance during locomotion or in more challenging conditions [31].

A possible shortcoming of (only) using the BIs proposed in BenchBalance might be the impossibility of differentiating between distinct balance strategies (e.g., hip or ankle strategies) [7]. As it was presented in the results of this manuscript, different courses in $q_{sway}$ are often associated with the use of distinct balance strategies, but the metric $q_{sway}$ does not include by itself information about the type of joint used to keep in balance. Moreover, the $q_{sway}$ and the recovery time do not quantify the human-likeness of the balance response. This evaluation of human-likeness might be interesting for future studies, since a wearable exoskeleton that behaves as a human could be perceived as more transparent and reliable by the user [4,30].

## 5. Conclusions

In this contribution, we presented the BenchBalance system, which is a portable benchmarking solution proposed to conduct reproducible balance assessments of wearable exoskeletons and their users. The two key elements of the system (a hand-held perturbator and a smart garment) have been described in detail, as well as the developed software interface and the algorithms used to perform the balance assessment.

A proof of principle of the BenchBalance system was carried out with a single participant in two case scenarios: participant without exoskeleton and participant wearing a lower-limb exoskeleton. It provided an indication of the functional capabilities of the system and the potential of the derived balance metrics.

### Future Work

The BenchBalance system has been developed for the EUROBENCH consortium and will be incorporated as a testbed at the EUROBENCH facility. The purpose of EUROBENCH is to test the performance of wearable exoskeletons at any stage of development and on a large scale. This will generate future large datasets of the assessment and comparison of balance capabilities of different wearable exoskeletons and/or their implemented controllers.

## Figures and Tables

**Figure A1:**
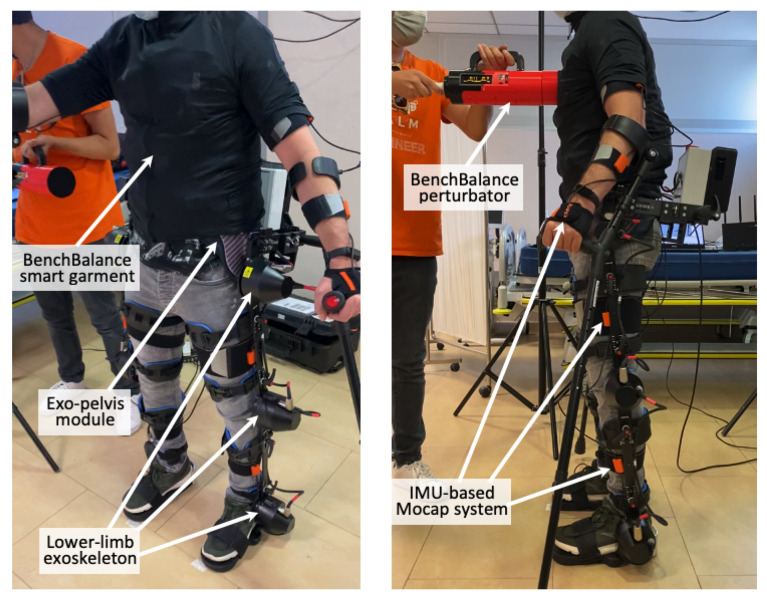
Setup for the use of BenchBalance together with a lower-limb exoskeleton and an IMU-based Mocap system.

## Data Availability

Not applicable.

## Supplementary Materials

The following are available online at https://www.mdpi.com/article/10.3390/s22010119/s1, Video S1: video material showing the subsystems of BenchBalance and the proof of principle with a healthy subject.

## Appendix A

In this appendix, we present the results of the BIs calculated for each of the sixteen conditions of the protocol and the two scenarios tested (participant without and with the exoskeleton).

**Table A1:** Mean and standard deviation of the calculated BIs considering the five good repetitions within each condition of the protocol. Two scenarios were tested: participant not wearing and wearing the exoskeleton.

	Without Exoskeleton		With Exoskeleton	
**Condition Order**	**Sway Angle (deg)**	**Recovery Time (s)**	**Sway Angle (deg)**	**Recovery Time (s)**
1	1.64±0.43	2.40±0.53	1.07±0.19	1.99±0.59
2	2.18±0.37	2.20±0.15	0.81±0.42	2.94±0.42
3	2.79±0.38	2.53±0.27	1.48±0.59	3.04±0.94
4	2.82±0.60	2.73±0.66	0.58±0.32	2.86±0.97
5	−1.93±0.86	2.84±0.12	−2.68±0.99	2.99±0.79
6	−1.31±0.38	2.74±0.28	−2.85±0.81	3.01±0.34
7	−2.41±0.24	2.74±0.40	−3.63±1.16	2.74±0.70
8	−2.29±0.85	3.29±0.79	−4.51±0.96	3.32±0.42
9	−1.05±0.62	2.01±0.63	−2.68±0.33	3.08±0.48
10	−0.90±0.16	1.85±0.10	−3.55±0.81	3.28±0.53
11	−2.64±0.68	3.23±0.73	−4.80±0.56	3.57±0.55
12	−2.31±0.31	3.59±0.51	−4.24±0.79	3.61±0.49
13	1.00±0.59	2.51±0.33	2.99±1.09	2.86±0.28
14	0.84±0.26	2.58±0.94	3.45±0.40	3.09±0.422
15	1.99±0.85	2.66±0.75	2.11±1.35	3.58±0.46
16	0.94±0.45	2.78±1.05	2.88±0.60	3.76±0.45

## Appendix B

The calculation of the CoM performed with the BenchBalance model was compared with a reference estimation retrieved from a commercial Motion capture system and its 3D full biomechanical model (Xsens MVN [28]). In the table below, we show the mean ± SD across all the frontal and sagittal conditions of the protocol for the linear correlation coefficient (Pearson’s correlation coefficient, *r*) and the root mean square error (RMSE) between the sway angle computed by both systems. To compute the mean ± SD for both planes, we used the data of this manuscript (including all sixteen protocol conditions) that referred to the scenario of the participant not wearing the exoskeleton. The RMSEs were calculated for the time interval 0.5 s before and 5 s after the perturbation onsets.

**Table A2:** Mean ± SD of the linear Pearson’s correlation coefficient (*r*) and RMSE for sagittal and frontal planes calculated between the sway angles ($q_{sway}$) computed with the BenchBalance model and with Xsens MVN system. Values averaged across all the frontal and sagittal conditions of the protocol.

Plane	BI	*r*	RMSE (deg)
Sagittal	$q_{sway-AP}$	0.9849 ± 0.0127	0.2556 ± 0.1692
Frontal	$q_{sway-ML}$	0.8897 ± 0.1014	0.4286 ± 0.2139

## Appendix C

In this appendix, we show the system setup during a test with a human wearing an exoskeleton at the EUROBENCH facilities (Figure A1). See also Video S1 in Supplementary Materials for a real demonstration of the use of the system.
